# Effects of a pulmonary rehabilitation program in a patient with very severe chronic obstructive pulmonary disease at a primary care center: A case report

**DOI:** 10.1016/j.jtumed.2025.12.009

**Published:** 2026-01-21

**Authors:** Mohammed E. Alsubaiei

**Affiliations:** Department of Physical Therapy, Faculty of Applied Medical Sciences, Imam Abdulrahman bin Faisal University, Dammam, KSA

**Keywords:** Chronic obstructive pulmonary disease, Primary care center, Pulmonaryrehabilitation

## Abstract

**Introduction:**

Severe chronic obstructive pulmonary disease (COPD) is characterized by dyspnea, frequent exacerbations, and diminished quality of life. For patients awaiting lung transplantation, managing symptoms and optimizing physical and psychological health are critical. Pulmonary rehabilitation (PR) is a comprehensive, evidence-based intervention designed to improve these outcomes through exercise training, education, and psychological support.

**Methods:**

This case report describes a 53-year-old man with very severe COPD (GOLD group E) who was referred to PR after he refused to undergo a lung transplant operation. At baseline, he presented with functional limitation (mMRC = 4, CAT = 35, 6MWD = 200 m), severe muscle weakness (3/5), and a high hospital anxiety and depression score (HADS = 20). A 12-week individualized PR program was implemented, consisting of weekly supervised high-intensity interval endurance and resistance training, inspiratory muscle training, and educational sessions including cognitive behavioral therapy (CBT), alongside a prescribed home exercise program.

**Results:**

After completion of the 12-week program, the patient showed substantial improvements across all measured parameters. His dyspnea significantly decreased (mMRC = 2), and his health status impact score markedly decreased (CAT = 8). His exercise capacity significantly improved, with a 6-min walk distance increase of 150 m (to 350 m) without supplemental oxygen. His muscle strength normalized to 5/5 in all limbs, and his psychological well-being showed remarkable recovery, with a HADS score improvments to 10, thus indicating amelioration of severe symptoms.

**Conclusion:**

This case illustrates that a structured PR program including CBT can provide physical and psychological benefits for patients with very severe COPD before lung transplantation. PR is an essential intervention to optimize patient status, improve quality of life, and potentially prepare individuals for future surgical interventions.

## الملخص

**أهداف البحث:**

يتميز مرض الانسداد الرئوي المزمن الحاد بضيق التنفس، ونوبات التفاقم المتكررة، وتدني جودة الحياة. بالنسبة للمرضى المنتظرين لزراعة الرئة، يُعدّ التحكم في الأعراض وتحسين الصحة البدنية والنفسية أمرًا بالغ الأهمية. إعادة التأهيل الرئوي هو تدخل شامل قائم على الأدلة، مصمم لتحسين هذه النتائج من خلال التدريب الرياضي، والتثقيف، والدعم النفسي.

**طرق البحث:**

يصف هذا التقرير حالة رجل يبلغ من العمر 53 عامًا مصاب بمرض الانسداد الرئوي المزمن الحاد جدا (المجموعة E وفقًا لتصنيف GOLD)، والذي أُحيل إلى برنامج إعادة التأهيل الرئوي بعد رفضه الخضوع لعملية زراعة الرئة. عند التقييم الأولي، كان يعاني من قصور وظيفي (مقياس ضيق التنفس المعدل=4، مقياس تقييم مرض الانسداد الرئوي المزمن=35، اختبار المشي لمدة 6 دقائق = 200 متر)، وضعف عضلي شديد (3/5)، ودرجة عالية من القلق والاكتئاب. تم تطبيق برنامج تأهيل رئوي فردي لمدة 12 أسبوعا، يتضمن تدريبات أسبوعية تحت إشراف متخصصين، تشمل تمارين التحمل والمقاومة عالية الكثافة، وتدريب عضلات الشهيق، وجلسات تثقيفية تتضمن العلاج السلوكي المعرفي، بالإضافة إلى برنامج تمارين منزلية موصوف.

**النتائج:**

بعد إتمام البرنامج، أظهر المريض تحسنا ملحوظا في جميع المؤشرات المقاسة. انخفض ضيق التنفس لديه بشكل كبير (مقياس ضيق التنفس المعدل = 2)، وانخفض مؤشر تأثير حالته الصحية بشكل ملحوظ (مقياس تقييم مرض الانسداد الرئوي المزمن = 8). تحسنت قدرته على ممارسة الرياضة بشكل ملحوظ، حيث زادت مسافة المشي لمدة 6 دقائق بمقدار 150 مترا (إلى 350 مترا) دون الحاجة إلى أكسجين إضافي. عادت قوة عضلاته إلى طبيعتها (5/5) في جميع الأطراف، وشهدت حالته النفسية تحسنا ملحوظا، حيث ارتفع مؤشر القلق والاكتئاب في مقياس هاملتون للاكتئاب إلى 10، مما يشير إلى تحسن الأعراض الشديدة.

**الاستنتاجات:**

توضح هذه الحالة أن برنامج التأهيل الرئوي المنظم، والذي يتضمن العلاج السلوكي المعرفي، يمكن أن يوفر فوائد بدنية ونفسية للمرضى الذين يعانون من مرض الانسداد الرئوي المزمن الشديد قبل زراعة الرئة. يُعد التأهيل الرئوي تدخلاً أساسياً لتحسين حالة المريض، وتحسين نوعية الحياة، وربما إعداد الأفراد للتدخلات الجراحية المستقبلية.

## Introduction

Severe chronic obstructive pulmonary disease (COPD) involves a marked decline in lung function due to airway obstruction^1^ and is typically diagnosed through spirometry readings indicating airflow obstruction, at <70 % of predicted values.^1^ Individuals with severe COPD experience breathlessness, which limits daily activities. This stage of the disease is often accompanied by increased risk of respiratory infections and chronic respiratory failure, in which the body struggles to maintain adequate oxygen and carbon dioxide levels.^1^ COPD is the fourth leading cause of death globally.^2^ Severe COPD poses a substantial burden to patients: it frequently leads to complications, emergency visits, and multiple hospital admissions, and it can decrease quality of life and increase mortality risk.^1^, ^2^, ^3^

Patients meeting specific criteria, often including severe hypoxemia, hypercapnia, and frequent exacerbations despite optimal medical management, may be eligible for lung transplantation as a last-resort intervention to improve survival and quality of life.^4^ However, patients who are eligible for lung transplantation frequently incur long wait times before a lung from a suitable donor becomes available, thus decreasing their quality of life.^5^ In KSA, only 80 lung transplants were performed from 2010 until 2015, and 37 were performed in 2017.^6^ Before lung transplantation, patients can be managed by pharmacological therapy and non-pharmacological therapy, such as pulmonary rehabilitation (PR), to ameliorate their symptoms and quality of life.^1^^,^^7^

PR is among the best options for the management of severe COPD and other respiratory diseases.^8^ This therapy includes a comprehensive program designed to increase exercise tolerance, decrease symptoms such as shortness of breath, and enhance overall quality of life for patients with severe COPD.^9^ This medically supervised program, typically delivered in an outpatient setting, includes various elements such as exercises, education, and psychological support to achieve these goals.^10^

Exercise training programs, a fundamental part of PR,^11^ include supervised sessions with healthcare professionals, with a focus on both aerobic endurance and strength training.^11^ Aerobic exercises, such as treadmill walking or stationary cycling, have been found to improve cardiovascular health and increase exercise capacity with improvement in oxygen consumption during exercise.^11^ Strength training with weights or resistance bands enhances muscle strength and endurance, and allows patients to perform daily activities with diminished shortness of breath.^11^ PR also includes inspiratory muscle training and breathing exercises. For example, pursed-lip breathing is a frequently used technique that helps patients manage shortness of breath during exertion and improve their overall breathing efficiency.^12^

In addition, PR programs offer education and support to increase patients' awareness of the nature of the disease and management options. Education can help patients change their behavior and lifestyle in a healthy manner.^13^ Patients learn about their specific lung condition, proper medication use, and disease management strategies that can help improve their quality of life.^13^ Educational sessions may also cover nutrition counseling, stress management, and smoking cessation, if applicable. Through this approach, PR empowers patients with COPD to take control of their health and live healthy lives.^13^

Furthermore, PR offers psychological benefits for patients with severe COPD.^13^ PR not only improves physical capacity and breathlessness, but also has been demonstrated to decrease the symptoms of anxiety and depression that are highly prevalent in this population.^13^ This effect is probably due to a comprehensive approach including cognitive behavioral therapy (CBT).^13^ In addition, education and self-management techniques allow patients to control their condition.^13^ Overall, PR has been demonstrated to be a powerful tool for enhancing mental well-being in patients with severe COPD.^11^, ^12^, ^13^

Beyond its benefits in managing the symptoms of severe COPD and improving physical capacity, PR plays a critical role in enhancing the condition of patients considered eligible for lung transplantation, by improving their physical and psychological condition.^4^^,^^14^ Because PR programs encourage patient adherence to postoperative exercise programs, they can help optimize lung transplantation outcomes.^14^ In conclusion, PR is an essential preoperative program for preparing patients, because it increases the likelihood of successful lung transplantation and decreases the risk of postoperative complications. In KSA, the provision of PR programs is limited by a lack of healthcare providers and funding.^15^ This case study illustrates the effects of a comprehensive rehabilitation program tailored for patients with severe COPD awaiting lung transplantation, and it additionally demonstrates the feasibility and benefits of conducting PR at a primary care center.

## Case report

This case report was approved by the local Institutional Review Board (IRB) at Imam Abdulrahman Bin Faisal University, Dammam, KSA (IRB number 2024-03-397). The patient provided a signed form consenting to the publication of this case report.

A 53-year-old man was diagnosed with severe COPD (severe emphysema) 3 years prior. He had a daily productive cough with sputum and shortness of breath, and he had been admitted to the hospital more than three times per year for COPD exacerbations. He previously smoked 100 pack-years. On examination, his chest showed signs of hyperinflation. He had normal heart sounds, with no murmurs or added sounds. His high-resolution computed tomography in 2017 showed bilateral severe centrilobular emphysema, which was more severe at the upper lobes, as well as right upper lobe linear fibrotic changes with calcification and traction bronchiectasis, which were suggestive of old granulomatous disease and bilateral multiple calcified nodules. His pulmonary function test (PFT) indicated very severe obstruction with no reversibility, his high total lung capacity suggested hyperinflation, and his high residual volume suggested air trapping. His PFT parameters were as follows: FEV1/FVC: 0.34 L (34 %), FEV1: 0.79 L (23 %), FVC: 2.28 L (53 %) residual volume: 6.30 L (289 %), and total lung capacity: 8.52 L (126 %). He was classified in COPD group E according to GOLD classification 2024.^16^ The patient's condition was discussed with a thoracic surgery team for consideration of lung volume reduction surgery. No specific target area was identified, because the emphysema covered a large area, and lung transplantation was therefore considered. However, the patient refused to undergo lung transplant surgery and was referred to a PR program.

In the first PR session, portable oxygen therapy was used when needed. We performed multiple assessments before the PR program to identify his baseline characteristics. His Modified Medical Research Council Dyspnea Scale (mMRC) score was 4, according to the 2024 GOLD classification.^16^ His score of 35 on the COPD Assessment Test (CAT) indicated very high health impact. His exercise capacity was assessed with a 6 min walking test (6MWT) according to American Thoracic Society guidelines.^7^ Pre- and post-rehabilitation tests were conducted on the same 25-m, flat, straight primary care center corridor at approximately the same time of day. To ensure consistency, the test was completed by the same cardio-pulmonary physical therapist. The patient walked 200 m with oxygen therapy in the last 2 min (nasal cannula with 2 L oxygen flow). For muscle power assessment, a manual muscle test was performed by a cardio-pulmonary physical therapist. The patient's positioning and the techniques used for applying resistance were strictly standardized and replicated in the post-intervention assessment. The results indicated 3/5 in both the upper limb and lower limb. Anxiety and depression were evaluated with the Hospital Anxiety and Depression Scale (HADS). His score of 20 indicated very severe depression and anxiety symptoms, and he had previously attempted suicide once.

For the PR program, we designed 12 weeks of exercises and an education program based on his baseline evaluation. The program involved one supervised session at a family and community center every week. In addition, home exercises, including walking and resistance exercises, were given to the patient. High-intensity interval training (HIIT) was used for endurance. The exercise intensities for aerobic exercise were based on heart rate monitoring with the following formula: target heart rate (THR) = (heart rate reserve × intensity %) + resting heart rate. The resistance exercises for both the upper and lower limbs were based on the one-repetition maximum. Both aerobic and resistance exercises started at low intensity (30 % of the baseline assessment), and the intensity was gradually increased every week until high intensity was reached (120 % of the baseline assessment). The program included walking on a treadmill, using arm ergometers, bicycling, and stepping exercises. For resistance exercise, weight exercises for the upper limbs (such as bicep curls, triceps extension, chest press, and shoulder exercises) and lower limbs (such as leg extension and flexion, and leg squat) were conducted in three sets with 15, 12, and 10 repetitions. At the end of the exercise session, inspiratory muscle training was conducted with a dedicated pressure-threshold device (30 breaths three times per session, with increased intensity every 2 weeks until maximum intensity was reached). During the exercise session, his oxygen saturation levels (92 %–88 %), heart rate, and blood pressure were monitored. The duration of each session was 1 h.

For the education program, 30-min one-on-one education sessions were conducted each week. Education regarding the disease, medication use, personal hygiene, and home exercises was provided to the patient. In addition, CBT was conducted every week by a professional health care provider with special training in using CBT techniques to improve behavior and change negative thoughts. The trained health care providers used Socratic questioning during the CBT session. All the patient's questions were answered during the education sessions. The duration of each education and CBT session was 1 h.

After 12 supervised PR sessions, the patient showed an improved mMRC score of 2. His HADS score was 10, his CAT score was 8, and he was able to walk 350 m in 6 min without stopping. His muscle power for the upper and lower limbs was 5/5, as measured with manual muscle testing. His oxygen saturation did not change, and ranged from 92 % to 88 %. No data on medication use changes were reported. In addition, the patient did not experience any exacerbations during the 12 weeks.

## Discussion

To our knowledge, this case report describes one of the first documented PR program services for a patient with COPD at a primary care center in KSA. This 53-year-old patient showed marked improvements in exercise capacity, shortness of breath, and mental health after completion of 12 weeks of once-weekly supervised sessions in the PR program. In addition, a comparison of the patient's improvements in the context of the broader evidence base for PR highlighted the role of PR in bridging gaps to transplant eligibility and enhancing quality of life in patients with severe COPD.

### Exercise capacity improvements

In this case report, the patient showed improvements in exercise capacity and muscle strength. His 6MWT distance improved from 200 m to 350 m, in alignment with meta-analysis data demonstrating PR's efficacy in improving exercise capacity (mean increase: 50–100 m).^7^ This result might have been due to the use of HIIT in our program, which has been demonstrated to enhance oxygen utilization and endurance in severe COPD.^11^^,^^17^ A meta-analysis of randomized controlled trials has reported that HIIT improves quality of life, exercise capacity, and pulmonary function in patients with COPD.^17^ Many studies have recommended that HIIT be included in exercise programs for patients with severe COPD.^17^ In addition, resistance training further contributed to our patient's increased muscle strength (3/5 to 5/5) and amelioration of sarcopenia, a common condition in severe COPD.^18^ These gains are critical for transplant candidates, because pre-transplant functional status predicts postoperative survival.^19^

### Symptom improvements

Our patient's decreased dyspnea (mMRC score decrease from 4 to 2) and CAT score (from 35 to 8) reflected the benefits of our PR program in symptom management. This finding was in line with those from previous studies indicating mMRC and CAT score improvements in patients with COPD.^20^ CAT score improvements reflect improvements in patients' symptoms and have been suggested to contribute to decreasing exacerbations and hospitalization.^1^^,^^8^^,^^17^ The improvement in our patient might be explained by our use of two strategies: (1) inspiratory muscle training, which has been demonstrated to improve CAT scores^21^ and (2) education on self-management and breathing techniques, such as pursed-lip breathing, as recommended by GOLD guidelines.^1^ The improvements in CAT and mMRC scores suggested the added value of individualized education and inspiratory muscle training, which has been demonstrated to improve respiratory mechanics^21^

### Psychological outcomes

The patient's HADS score decreased from 20 (indicating clinical anxiety or depression) to 10 (indicating borderline abnormality). The integrated CBT is likely to explain these effects. Many studies have reported that integrating CBT into PR programs significantly enhances both psychological and physical outcomes.^21^, ^22^, ^23^, ^24^ Recent evidence has confirmed that CBT, when delivered as a core component of multidisciplinary PR, effectively decreases symptoms of anxiety and depression, which are highly prevalent in this patient population.^19^, ^20^, ^21^ This psychological support helps patients better manage dyspnea, fear, and negative thinking, and consequently improves adherence to exercise regimens and more effective self-management strategies.^21^, ^22^, ^23^, ^24^ As a result, patients experience notable improvements in exercise capacity and health-related quality of life, and decreased hospital readmission rates; consequently, addressing the cognitive and behavioral aspects of breathlessness is key to optimizing the benefits of rehabilitation in severe COPD.^22^, ^23^, ^24^ CBT is therefore a recommended addition to PR programs for patients with COPD.

### Primary care delivery model

Our PR program was based on primary care centers, which might offer substantial advantages over traditional hospital-based programs for patients with severe COPD, by enhancing accessibility and long-term adherence. Although hospital-based PR is effective, many barriers, such as travel burden and limited availability, might potentially influence PR adherence and dropout rates.^8^ In contrast, primary care-based PR decreases travel time, and makes participation more feasible and less stressful for patients with COPD, because primary care centers are easily accessible.^11^ Many studies have suggested that PR delivered in this familiar, local setting is as effective as hospital PR programs in improving patient outcomes such as exercise capacity, dyspnea, and health-related quality of life.^25^ Furthermore, the primary care model facilitates continuity of care to manage common comorbidities in severe COPD.^26^^,^^27^ Primary care centers support a more approach to disease management and promote long-term maintenance of PR benefits within the patient's environment, thus ultimately providing a more sustainable and patient-centered option.^8^^,^^26^^,^^27^

### Limitations and future directions

This case highlights the feasibility of a comprehensive, multidisciplinary PR model combining HIIT, resistance training, inspiratory muscle training, and CBT. However, long-term follow-up was not performed, and the patient's motivation might have influenced the outcomes. Larger trials are needed to validate this program in transplant-eligible patients.

Although this case study suggests that once-weekly PR sessions were beneficial for our patient with severe COPD awaiting lung transplantation in a primary care setting, this finding is difficult to generalize to all patients. However, the results might provide a rationale for larger-scale studies to test the feasibility of decreasing the recommended number of sessions per week. Implementing fewer sessions per week might potentially lower treatment costs and increase program enrollment, thereby making PR more accessible.

Our patient's marked improvements in functional capacity and muscle strength must be interpreted with caution, because they exceeded the average response reported in patients with very severe COPD undergoing PR. The remarkable changes observed might potentially be explained by our patient's high motivation. An additional limitation is that no control group was included. The intensive one-on-one program and low baseline measurements might have enabled these substantial changes in outcomes. Therefore, the findings should not be generalized without further investigation in larger studies.

An additional limitation of this case report is the absence of post-rehabilitation PFT data. Therefore, we cannot comment on any potential physiological changes in lung function resulting from the intervention.

## Conclusion

This case report demonstrated PR's benefits in managing a patient with severe COPD. PR not only improves physical activity, symptom control, quality of life, and mental health, but also prepares individuals for potential future transplantation. Clinicians should recommend early, tailored PR in advance of transplantation for patients with COPD, and psychological support should be integrated to overcome negative thoughts in this population.
